# Nonlocal Strain Gradient Model for the Nonlinear Static Analysis of a Circular/Annular Nanoplate

**DOI:** 10.3390/mi14051052

**Published:** 2023-05-15

**Authors:** Mostafa Sadeghian, Arvydas Palevicius, Giedrius Janusas

**Affiliations:** Faculty of Mechanical Engineering and Design, Kaunas University of Technology, Studentu 56, 51424 Kaunas, Lithuania

**Keywords:** nonlinear static analysis, circular/annular nanoplate, nonlocal strain gradient theory, FSDT, HSDT, DQM

## Abstract

A nonlinear static analysis of a circular/annular nanoplate on the Winkler–Pasternak elastic foundation based on the nonlocal strain gradient theory is presented in the paper. The governing equations of the graphene plate are derived using first-order shear deformation theory (FSDT) and higher-order shear deformation theory (HSDT) with nonlinear von Karman strains. The article analyses a bilayer circular/annular nanoplate on the Winkler–Pasternak elastic foundation. HSDT while providing a suitable distribution of shear stress along the thickness of the FSDT plate, eliminating the defects of the FSDT and providing good accuracy without using a shear correction factor. To solve the governing equations of the present study, the differential quadratic method (DQM) has been used. Moreover, to validate numerical solutions, the results were compared with the results from other papers. Finally, the effect of the nonlocal coefficient, strain gradient parameter, geometric dimensions, boundary conditions, and foundation elasticity on maximum non-dimensional deflection are investigated. In addition, the deflection results obtained by HSDT have been compared with the results of FSDT, and the importance of using higher-order models has been investigated. From the results, it can be observed that both strain gradient and nonlocal parameters have significant effects on reducing or increasing the dimensionless maximum deflection of the nanoplate. In addition, it is observed that by increasing load values, the importance of considering both strain gradient and nonlocal coefficients in the bending analysis of nanoplates is highlighted. Furthermore, replacing a bilayer nanoplate (considering van der Waals forces between layers) with a single-layer nanoplate (which has the same equivalent thickness as the bilayer nanoplate) is not possible when attempting to obtain exact deflection results, especially when reducing the stiffness of elastic foundations (or in higher bending loads). In addition, the single-layer nanoplate underestimates the deflection results compared to the bilayer nanoplate. Because performing the experiment at the nanoscale is difficult and molecular dynamics simulation is also time-consuming, the potential application of the present study can be expected for the analysis, design, and development of nanoscale devices, such as circular gate transistors, etc.

## 1. Introduction

Due to their high stiffness and strength; elastic modulus (higher than 1 TPa); weight ratio; and also outstanding mechanical, electrical, and chemical properties, nanostructures have been applied in diverse domains, including nanoelectromechanical systems, as their high sensitivity and low mass make them perfect for demands in different areas. such as biosensors, medicine, computers, etc. [1,2,3].

The local elasticity model cannot prognosticate extremely small-sized effects on nanostructures because of the deficiency in the nonlocal elasticity model. Because the material’s length scale lacks a nonlocal parameter, the classical elasticity model cannot predict the minuscule size role of nanostructures. Experiments and molecular dynamics simulations are time-consuming, high in cost, and laborious with respect to analyzing nanostructure systems. Diverse theories of nonlocal elasticity, such as Eringen (nonlocal elasticity), strain gradient, couple stress, and surface stress, have been utilized to make atypical plate models in order to overcome this problem [4,5]. Using nonlocal elasticity theory, Su and Zhou [6] perused the electromechanical coupling responses of flexoelectric nanostructures.

Recently, many researchers have focused on the mechanical behavior of structures based on the nonlocal strain gradient theory [7,8,9]. For example, Penna et al. [10] studied the bending analysis of porous FG nanobeams using the nonlocal strain gradient theory. In another study, the nonlocal strain gradient model was used by Le et al. [11] for examining static and vibration investigations of the FG sandwich nanoplate. Karami and his colleagues [12] employed the nonlocal strain gradient theory to analyze functionally graded anisotropic nanoplates. Alghanmi [13] studied the bending of functionally graded porous nanoplates using the nonlocal strain gradient model and concluded that deflection is enhanced by reducing the length scale parameter. Gui and Wu [7] examined the buckling of nanoshells exposed to the axial load by considering thermal, magnetic, and electric conditions. Arefi et al. [14] perused the investigation of the bending of a porous sandwich nanoplate with piezomagnetic properties based on the theory of nonlocal strain gradients.

It is known that the classical theory (which neglects shear effects) is the simplest model for analyzing structures such as beams and plates [15]. However, this theory underestimates deflection and overestimates natural frequencies/critical buckling loads. In this regard, the FSDT was proposed, which provides more accurate results than the former theory, and many researchers have used it in their analyses [16,17]. Using FSDT, Nguyen and Phung [18] investigated the bending, buckling, and vibration of FGM plates. Padawale et al. [19] examined the vibration of the annular plate (exposed to a temperature at one edge) according to the FSDT. Qin et al. [20] investigated the bending and vibrations of circular plates based on FSDT. He and his colleagues [21] perused the bending and free vibration examination of ribbed plates based on FSDT.

However, FSDT needs a shear correction coefficient to rectify stress-free boundary conditions. To solve this problem, the HSDT was proposed, and many researchers have employed it in their analyses [15,22,23]. For example, Pavan et al. studied the statics, buckling, and free vibration of composite beams using HSDT [24]. Rodrigues [25] perused the bending of cross-ply laminates based on HSDT and a radial point interpolation technique. Zghal and Dammak [26] perused the vibration of plates and shells with functionally graded pores imperfections based on FSDT using the finite element method. Using modified FSDT, Trabelsi et al. [27] perused the thermal post-buckling of functionally graded material structures. Using an improved FSDT, Zghal et al. [28] analyzed the thermal-free vibrations of functionally graded plates and panels. Based on FSDT, Zghal et al. [29] investigated the functionality of graded carbon-nanotube-reinforced composite structures using the finite element technique. Zghal et al. [30] carried out an analysis of the nonlinear bending of graphene-nanotube-reinforced nanocomposites by utilizing the finite shell element and membrane enhancement, which includes a high-order variation of the displacement field.

Aghababaei and Reddy [31] utilized a nonlocal third-order shear deformation model to analytically study the vibration-free bending of rectangular plates under a simply supported boundary condition in order to obtain the natural frequency and deflection of plates.

Recently, advanced theories for the nonlinear examination of functionally graded carbon-nanotube-reinforced composite plates and shells have been studied [32,33,34]. For example, Zghal et al. [35] performed a static linear examination of functionally graded carbon-nanotube-reinforced plates and shells. In their paper, they utilized a micromechanical model (extended rule of mixture) to define material properties. Frikha et al. [36] investigated the nonlinear bending of functionally graded carbon-nanotube-reinforced composite shells by utilizing the Kirchhoff shell model. Zghal et al. [37] carried out research on the post-buckling of functionally graded carbon-nanotube-based structures. Zghal et al. [38] investigated the buckling of functionally graded carbon-nanotube-reinforced composite structures by employing a double director finite element shell theory.

The vibration behavior of size-dependent structures using the non-local-strain gradient theory or Eringen’s theory has been presented in some studies. For example, Qaderi et al. [39] studied cracked graphene-platelet-reinforced composite plates subjected to parametric excitation based on Eringen’s theory and the Line spring model. Mahinzare et al. [40] studied the vibration of magnetically actuated viscoelastic functionally graded nanoshells based on the nonlocal strain gradient theory and FSDT. Rashidpour et al. [41] perused the dynamic analysis of the viscoelastic laminated composite nanoplate using the nonlocal strain gradient theory and FSDT, applying the Galerkin method. Ghorbani et al. [42] studied surface effects on the natural frequency of a functionally graded cylindrical nanoshell using the nonlocal strain gradient theory via generalized DQM.

DQM is a powerful numerical method that has been used in many papers [43,44]. The basic goal of this technique is to apply Lagrange interpolation polynomials to field coefficients and to solve the equations at discrete grid points. Improved accuracies can be achieved by using more grid points. Han et al. [45] employed DQM to examine one-electrode micro-resonators using a generalized 1-DOF model. Liu et al. [46] used DQM to analyze the bending of FGM sandwich plates by considering the tunable auxetic core.

To our best knowledge, FSDT and HSDT with the nonlocal strain gradient theory for the static analysis of circular/annular nanoplates via DQM have not been employed. Furthermore, this paper analyzed a bilayer circular/annular nanoplate located on elastic foundations. The effects of the non-local coefficient, strain gradient, geometric dimensions, boundary conditions, and foundation elasticity on the results of the maximum nondimensional deflection are investigated. Moreover, the deflection results obtained by HSDT have been compared with the results of FSDT, and the importance of using higher-order models has been highlighted. From the results, it can be concluded that both the strain gradient parameter and the nonlocal coefficient have notable effects with respect to reducing (or enhancing) the dimensionless maximum deflection. Additionally, replacing a bilayer nanoplate (considering van der Waals forces between layers) with a single-layer nanoplate (with the same equivalent thickness) is not possible when obtaining exact deflection results. In addition, the deflection of the single-layer nanoplate is lower than the deflection of a bilayer nanoplate. At higher thickness-to-radius ratios, it is better to use HSDT to obtain more accurate results than FSDT. As performing the experiment is difficult because a nanoscale and molecular dynamics simulation is time-consuming, potential applications of the present study can be expected for the analysis and design of nanoscale devices. The results of this paper can be useful for the development of nanostructured devices, such as circular gate transistors, etc.

## 2. The Governing Equations

### 2.1. The Governing Equations for the Axisymmetric Single-Layer Circular/Annular Nanoplate

**Figure 1:**
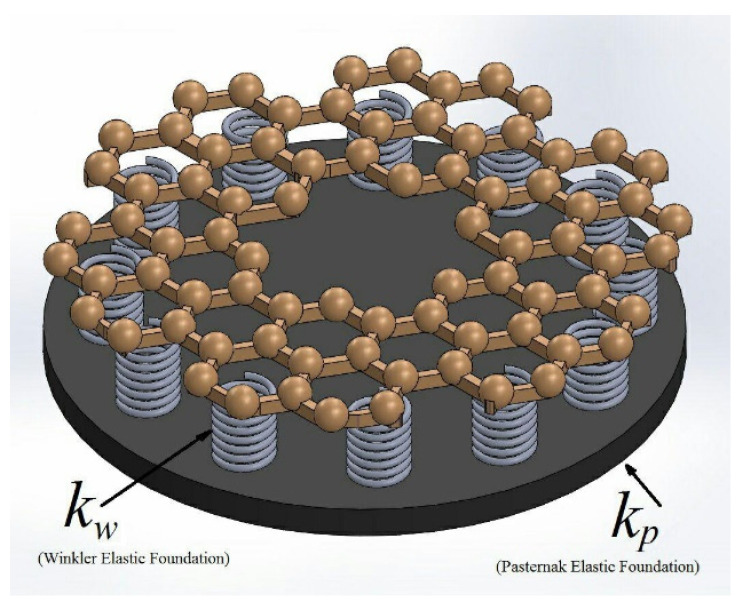
The graphene annular plate on elastic foundations.

**Figure 2:**
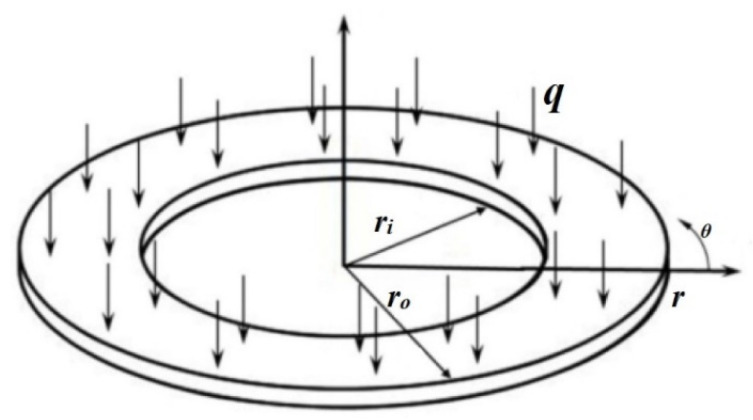
The annular plate under bending loads.

In this section, equilibrium equations have been derived by employing the energy method for the nonlinear analysis of single-layer circular/annular nanoplates under bending. To derive the equilibrium equations by the energy method, a displacement field using the HSDT and the nonlinear strain components with von Karman’s assumptions have been considered. Figure 1 demonstrates a graphene annular plate on the Winkler (*k_w_*) and Pasternak (*k_p_*) elastic foundations with *r_i_* (internal radius) and *r_o_* (outer radius). Additionally, Figure 2 shows the schematic of the annular plate under bending loads.

Taking into account the HSDT and adding a special function called *g*(*z*), the displacement field will be in the form of the following equations which are in the *r*, *θ*, and *z* directions defined by *U*, *V*, and *W*, respectively.

(1) $$\begin{array}{l} U(r,\theta ,z)=u_{0}(r)-z\frac{dw_{0}(r)}{dr}+g(z)\phi (r)\\ V(r,\theta ,z)=0\\ W(r,\theta ,z)=w_{0}(r) \end{array}$$

*u_0_*, *v_0_*, and *w_0_* are the displacement components of the midplane in the *r*, *θ*, and *z* directions, respectively. Also, *ϕ* and *ψ* are the rotation components around the *θ* and *r* axes, respectively. The function *g*(*z*) is defined as follows.

(2) $$g(z)=f(z)+zy^{*}$$

*f* (*z*) and y* are considered different functions used in various references and are listed in Table 1 (for example, the Ambartsumian [47] model can be considered as $\underset{f(z)}{\underset{︸}{-\frac{1}{6}z^{3}}}+\underset{y^{*}}{\underset{︸}{\frac{h^{2}}{8}}}z$).

The nonlinear strain components, taking into account von Karman’s assumptions, are as follows.

(3) $$\varepsilon_{r}=\frac{dU}{dr}+\frac{1}{2}{\left(\frac{dW}{dr}\right)}^{2}=\frac{du_{0}}{dr}-z\frac{d^{2}w_{0}}{dr^{2}}+g\left(z\right)\frac{d\phi }{dr}+\frac{1}{2}{\left(\frac{dw_{0}}{dr}\right)}^{2}$$

(4) $$\varepsilon_{\theta }=\frac{U}{r}=\frac{1}{r}\left(u_{0}-z\frac{dw_{0}}{dr}+g\left(z\right)\phi \right)$$

(5) $$\gamma_{r\theta }=0$$

(6) $$\gamma_{rz}=\frac{dW}{dr}+\frac{dU}{dz}=\phi \frac{dg\left(z\right)}{dz}$$

(7) $$\gamma_{\theta z}=0$$

The force and momentum resultants in the nonlocal form (*NL*) are defined as follows:

(8) $${\left\{N_{r},N_{\theta },Q_{r}\right\}}^{NL}=\int_{-\frac{h}{2}}^{\frac{h}{2}}{\left\{\sigma_{r},\sigma_{\theta },\sigma_{rz}\right\}}^{NL}dz$$

(9) $${\left\{M_{r},M_{\theta }\right\}}^{NL}=\int_{-\frac{h}{2}}^{\frac{h}{2}}{\left\{\sigma_{r},\sigma_{\theta }\right\}}^{NL}zdz$$

(10) $${\left\{R_{r},R_{\theta }\right\}}^{NL}=\int_{-\frac{h}{2}}^{\frac{h}{2}}{\left\{\sigma_{r},\sigma_{\theta }\right\}}^{NL}f\left(z\right)dz$$

(11) $$R_{rz}^{NL}=\int_{-\frac{h}{2}}^{\frac{h}{2}}\sigma_{rz}^{NL}f^{\prime }\left(z\right)dz$$

### 2.2. Derivation of Equilibrium Equations Based on the Energy Method

The potential energy of the system can be defined as the sum of the strain energy caused by the work of internal forces and the potential energy caused by external forces:

(12) Π=U+Ω

Π is the potential energy of the entire system, *U* is the strain energy of the system, and Ω is the potential energy of external forces. According to the principle of minimum potential energy, for a system in equilibrium, the variation in the potential energy is zero:

(13) δΠ=δU+δΩ=0

To write the variations of the strain energy of the system, the integral over the volume of strain energy density should be obtained. The strain energy density is as follows:

(14) $$\delta u_{v}=\sigma_{ij}\delta \varepsilon_{ij}$$

Therefore, for strain energy variations, we have the following:

(15) $$\delta U=\underset{V}{∭}\delta u_{v}dV=\underset{V}{∭}\sigma_{ij}\delta \varepsilon_{ij}dV$$

Therefore,

(16) $$\delta U=\underset{V}{∭}(\sigma_{r}\delta \varepsilon_{r}+\sigma_{\theta }\delta \varepsilon_{\theta }+\sigma_{rz}\delta \gamma_{rz})dV$$

Moreover,

(17) $$\delta \Omega =-\iint_{A}\left(q-k_{w}w_{0}+k_{p}\nabla^{2}w_{0}\right)r\delta w_{0}drd\theta$$

where *k_w_* and *k_p_* illustrate the Winkler and Pasternak elastic foundation coefficients, respectively. Therefore,

(18) $$\delta \Pi =\delta \Omega +\delta U=-\int_{0}^{2\pi }\int_{0}^{r}\left(q-k_{w}w_{0}+k_{p}\nabla^{2}w_{0}\right)r\delta w_{0}drd\theta +\delta U=0$$

By setting δΠ to zero, the coefficients of $\delta u_{0},\delta w_{0}$, and δϕ should be zero, and the Euler–Lagrange equations are obtained as follows. All results are nonlocal. Therefore, they are denoted with superscript *NL*.

(19) $$\delta u_{0}:N_{r}^{NL}+r\frac{dN_{r}^{NL}}{dr}-N_{\theta }^{NL}=0$$

(20) $$\begin{array}{l} \delta w_{0}:r\frac{d^{2}M_{r}^{NL}}{dr^{2}}+2\frac{dM_{r}^{NL}}{dr}-\frac{dM_{\theta }^{NL}}{dr}+\frac{dw_{0}}{dr}\left(N_{r}^{NL}+\frac{dN_{r}^{NL}}{dr}r\right)+rN_{r}^{NL}\frac{d^{2}w_{0}}{dr^{2}}+\\ \left(q-k_{w}w_{0}+k_{p}\nabla^{2}w_{0}\right)r=0 \end{array}$$

(21) $$\delta \phi :y^{*}\left(r\frac{dM_{r}^{NL}}{dr}+M_{r}^{NL}-M_{\theta }^{NL}-rQ_{r}^{NL}\right)+R_{r}^{NL}+r\frac{dR_{r}^{NL}}{dr}-R_{\theta }^{NL}-rR_{rz}^{NL}=0$$

Additionally, the third equilibrium equation can be written as follows:

(22) $$\begin{array}{l} \delta w_{0}:r\frac{d^{2}M_{r}^{NL}}{dr^{2}}+2\frac{dM_{r}^{NL}}{dr}-\frac{dM_{\theta }^{NL}}{dr}+\frac{dw_{0}}{dr}N_{\theta }^{NL}+rN_{r}^{NL}\frac{d^{2}w_{0}}{dr^{2}}\\ +\left(q-k_{w}w_{0}+k_{p}\nabla^{2}w_{0}\right)r=0 \end{array}$$

The theory of the nonlocal strain gradient (which can be considered as a combination of the nonlocal stress field and strain gradient model) has been derived by Lim et al. [54] and can be expressed as follows:

(23) $$(1-\mu^{2}\nabla^{2})\sigma_{ij}=C_{ijkl}(1-l^{2}\nabla^{2})\varepsilon_{kl},\nabla^{2}=\frac{d^{2}}{dr^{2}}+\frac{1}{r}\frac{d}{dr}$$

It should be noted that in Equation (23), $\mu ,C_{ijkl}$, and l signify nonlocal, elastic, and strain gradient (or internal material length scale) coefficients, respectively. Furthermore, the stress–strain constitutive equation in the nanoscale can be illustrated as follows [55]:

(24) $$\begin{array}{l} (1-\mu^{2}\nabla^{2})\left[\begin{array}{l} \sigma_{r}\\ \sigma_{\theta }\\ \sigma_{rz} \end{array}\right]=(1-l^{2}\nabla^{2})\left[\begin{array}{ccc} Q_{11} & Q_{12} & 0\\ Q_{12} & Q_{22} & 0\\ 0 & 0 & G_{13} \end{array}\right]\left[\begin{array}{l} \varepsilon_{r}\\ \varepsilon_{\theta }\\ \gamma_{rz} \end{array}\right],\;\\ \left\{\begin{array}{l} Q_{11}=\frac{E_{1}}{1-\nu_{12}\nu_{21}}\;,\;Q_{22}=\frac{E_{2}}{1-\nu_{12}\nu_{21}}\\ Q_{12}=\frac{\nu_{12}E_{2}}{1-\nu_{12}\nu_{21}}\;,\;G_{13}=\frac{E_{1}}{2(1+v_{12})} \end{array}\right. \end{array}$$

It is noted that in Equation (24), $E_{1}$ and $E_{2}$ denote the Young’s modulus along the 1 and 2 directions. Additionally, $v_{12}$ and $v_{21}$ are Poisson’s ratio in the mentioned directions, and $G_{13}$ is the shear modulus.

The nonlocal form is described as follows:

(25) $$\left(1-\mu \nabla^{2}\right){\left\{N_{r},N_{\theta },Q_{r}\right\}}^{NL}=\int_{-\frac{h}{2}}^{\frac{h}{2}}\left(1-\mu \nabla^{2}\right){\left\{\sigma_{r},\sigma_{\theta },\sigma_{rz}\right\}}^{NL}dz$$

The force and moment resultants in the local form are as follows:

(26) $${\left\{N_{r},N_{\theta },Q_{r}\right\}}^{L}=\int_{-\frac{h}{2}}^{\frac{h}{2}}{\left\{\sigma_{r},\sigma_{\theta },\sigma_{rz}\right\}}^{L}dz$$

(27) $${\left\{M_{r},M_{\theta }\right\}}^{L}=\int_{-\frac{h}{2}}^{\frac{h}{2}}{\left\{\sigma_{r},\sigma_{\theta }\right\}}^{L}zdz$$

(28) $${\left\{R_{r},R_{\theta }\right\}}^{L}=\int_{-\frac{h}{2}}^{\frac{h}{2}}{\left\{\sigma_{r},\sigma_{\theta }\right\}}^{L}f\left(z\right)dz$$

(29) $$R_{rz}{}^{L}=\int_{-\frac{h}{2}}^{\frac{h}{2}}\sigma_{rz}{}^{L}f^{\prime }\left(z\right)dz$$

Additionally, the resultants in terms of displacements are obtained in the following forms:

(30) $$\begin{array}{l} N_{r}^{L}=(1-l^{2}\nabla^{2})\{\frac{1}{1-\nu_{12}\nu_{21}}\left(E_{1}h\right.\left(\frac{du_{0}}{dr}+\frac{1}{2}{\left(\frac{dw_{0}}{dr}\right)}^{2}\right)+\nu_{12}E_{2}h\frac{1}{r}u_{0}\\ +\left(E_{1}\frac{d\phi }{dr}+\nu_{12}E_{2}\frac{1}{r}\phi \right)\int_{-\frac{h}{2}}^{\frac{h}{2}}\left.f\left(z\right)dz\right)\} \end{array}$$

(31) $$\begin{array}{l} N_{\theta }^{L}=(1-l^{2}\nabla^{2})\{\frac{1}{1-\nu_{12}\nu_{21}}\left(\nu_{12}E_{2}h\right.\left(\frac{du_{0}}{dr}+\frac{1}{2}{\left(\frac{dw_{0}}{dr}\right)}^{2}\right)+E_{2}h\frac{1}{r}u_{0}+\\ \left(\nu_{12}E_{2}\frac{d\phi }{dr}+E_{2}\frac{1}{r}\phi \right)\int_{-\frac{h}{2}}^{\frac{h}{2}}\left.f\left(z\right)dz\right)\} \end{array}$$

(32) $$\begin{array}{l} M_{r}^{L}=(1-l^{2}\nabla^{2})\{\frac{1}{1-\nu_{12}\nu_{21}}\left(E_{1}\right.\frac{h^{3}}{12}\left(-\frac{d^{2}w_{0}}{dr^{2}}+y^{*}\frac{d\phi }{dr}\right)+\\ \nu_{12}E_{2}\frac{h^{3}}{12}\left(-\frac{1}{r}\frac{dw_{0}}{dr}+y^{*}\frac{1}{r}\phi \right)+\left(E_{1}\frac{d\phi }{dr}+\nu_{12}E_{2}\frac{1}{r}\phi \right)\int_{-\frac{h}{2}}^{\frac{h}{2}}\left.zf\left(z\right)dz\right)\} \end{array}$$

(33) $$\begin{array}{l} M_{\theta }^{L}=(1-l^{2}\nabla^{2})\{\frac{E_{2}}{1-\nu_{12}\nu_{21}}\left(\nu_{12}\right.\frac{h^{3}}{12}\left(-\frac{d^{2}w_{0}}{dr^{2}}+y^{*}\frac{d\phi }{dr}\right)+\\ \frac{h^{3}}{12}\left(-\frac{1}{r}\frac{dw_{0}}{dr}+y^{*}\frac{1}{r}\phi \right)+\left(\nu_{12}\frac{d\phi }{dr}+\frac{1}{r}\phi \right)\int_{-\frac{h}{2}}^{\frac{h}{2}}z\left.f\left(z\right)dz\right)\} \end{array}$$

(34) $$\begin{array}{l} R_{r}^{L}=(1-l^{2}\nabla^{2})\{\frac{1}{1-\nu_{12}\nu_{21}}\left(\left(E_{1}\left(\frac{du_{0}}{dr}+\frac{1}{2}{\left(\frac{dw_{0}}{dr}\right)}^{2}\right)+\nu_{12}E_{2}\frac{1}{r}u_{0}\right)\right.\int_{-\frac{h}{2}}^{\frac{h}{2}}f(z)dz\\ +\left(\nu_{12}E_{2}\left(-\frac{1}{r}\frac{dw_{0}}{dr}+y^{*}\frac{1}{r}\phi \right)+E_{1}\left(-\frac{d^{2}w_{0}}{dr^{2}}+y^{*}\frac{d\phi }{dr}\right)\right)\int_{-\frac{h}{2}}^{\frac{h}{2}}zf(z)dz+\\ \left(E_{1}\frac{d\phi }{dr}+\nu_{12}E_{2}\frac{1}{r}\phi \right)\left.\int_{-\frac{h}{2}}^{\frac{h}{2}}{\left(f(z)\right)}^{2}dz\right)\} \end{array}$$

(35) $$\begin{array}{l} R_{\theta }^{L}=(1-l^{2}\nabla^{2})\{\frac{E_{2}}{1-\nu_{12}\nu_{21}}\left(\left(\nu_{12}\left(\frac{du_{0}}{dr}+\frac{1}{2}{\left(\frac{dw_{0}}{dr}\right)}^{2}\right)+\frac{1}{r}u_{0}\right)\right.\int_{-\frac{h}{2}}^{\frac{h}{2}}f(z)dz+\\ \left(\nu_{12}\left(-\frac{d^{2}w_{0}}{dr^{2}}+y^{*}\frac{d\phi }{dr}\right)+\left(-\frac{1}{r}\frac{dw_{0}}{dr}+y^{*}\frac{1}{r}\phi \right)\right)\int_{-\frac{h}{2}}^{\frac{h}{2}}zf(z)dz+\\ \left(\nu_{12}\frac{d\phi }{dr}+\frac{1}{r}\phi \right)(\int_{-\frac{h}{2}}^{\frac{h}{2}}{\left(f(z)\right)}^{2}dz)\} \end{array}$$

(36) $$Q_{r}^{L}=(1-l^{2}\nabla^{2})\{G_{13}\phi y^{*}h+G_{13}\phi \int_{-\frac{h}{2}}^{\frac{h}{2}}\left(f^{\prime }(z)+y^{*}\right)dz\}$$

(37) $$R_{rz}^{L}=(1-l^{2}\nabla^{2})\{G_{13}\phi \int_{-\frac{h}{2}}^{\frac{h}{2}}{\left(f^{\prime }\left(z\right)\right)}^{2}dz+G_{13}y^{*}\phi \int_{-\frac{h}{2}}^{\frac{h}{2}}f^{\prime }\left(z\right)dz\}$$

The equilibrium equations for a single-layer axisymmetric annular/circular nanoplate on a Winkler–Pasternak elastic foundation are locally expressed in the form of the following equations:

(38) $$\delta u_{0}:N_{r}^{L}+r\frac{dN_{r}^{L}}{dr}-N_{\theta }^{L}=0$$

(39) $$\begin{array}{l} \delta w_{0}:r\frac{d^{2}M_{r}^{L}}{dr^{2}}+2\frac{dM_{r}^{L}}{dr}-\frac{dM_{\theta }^{L}}{dr}+\left(1-\mu \nabla^{2}\right)\left(\left(q-k_{w}w_{0}+k_{p}\nabla^{2}w_{0}\right)r\right.\\ +N_{\theta }^{L}\frac{dw_{0}}{dr}+\left.rN_{r}^{L}\frac{d^{2}w_{0}}{dr^{2}}\right)+\mu r\left(\left(\nabla^{2}N_{r}^{L}\right)\frac{d^{2}w_{0}}{dr^{2}}+\left.\left(\nabla^{2}N_{\theta }^{L}\right)\left(\frac{1}{r}\frac{dw_{0}}{dr}\right)\right)\right.=0 \end{array}$$

(40) $$\delta \phi :y^{*}\left(r\frac{dM_{r}^{L}}{dr}+M_{r}^{L}-M_{\theta }^{L}-rQ_{r}^{L}\right)+R_{r}^{L}+r\frac{dR_{r}^{L}}{dr}-R_{\theta }^{L}-rR_{rz}^{L}=0$$

### 2.3. Equilibrium Equations of the Bilayer Axisymmetric Circular/Annular Nanoplate

**Figure 3:**
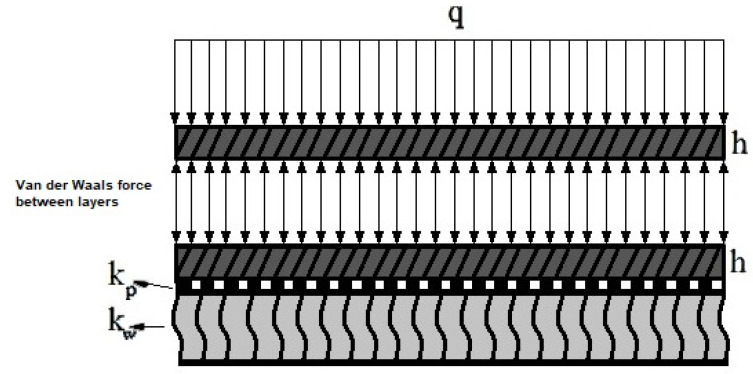
A bilayer nanoplate under bending loads on the Winkler–Pasternak elastic foundation considering the van der Waals force between the layers.

Graphene sheets have low bending strengths, and to solve this issue, several layers of graphene can be used. In this way, graphene sheets are placed on top of each other and are connected via weak van der Waals bonds, creating layers of graphene [47]. Figure 3 shows the schematic of the bilayer nanoplate under bending load (*q*) on the elastic foundations. A circular bilayer plate with radius *r_o_* and constant thickness *h* is considered. *k*_0_ is the van der Waals stiffness coefficient between two layers, and *kw* and *k_p_* are the coefficients of the Winkler and Pasternak elastic foundations, respectively.

Equilibrium equations for the bilayer circular/annular nanoplate on the Winkler–Pasternak elastic foundation are obtained almost in the same way as for the single-layer nanoplate. The displacement fields for the axisymmetric bilayer circular/annular nanoplate are as follows: index *i* = 1 represents the first layer, and *i* = 2 represents the second layer.

(41) $$U_{i}(r,\theta ,z)=u_{i}(r)-z\frac{dw_{i}(r)}{dr}+g\left(z\right)\phi_{i}(r)\;,\;i=1,2$$

(42) $$V_{i}(r,\theta ,z)=0\;,\;i=1,2$$

(43) $$W_{i}(r,\theta ,z)=w_{i}(r)\;,\;i=1,2$$

The strain equations are similar to those obtained for the single-layer circular/annular nanoplate. Only the energy equation, when using the minimum potential energy principle to obtain the equilibrium equations and boundary conditions for the upper and lower layers, is different according to the following equations:

(44) $$\delta \Omega_{1}=\int_{r_{i}}^{r_{o}}\int_{0}^{\tau }\left(q+k_{o}\left(w_{2}-w_{1}\right)\right)\delta w_{1}rdrd\theta$$

(45) $$\delta \Omega_{2}=\int_{r_{i}}^{r_{o}}\int_{0}^{\tau }\left(-k_{o}\left(w_{2}-w_{1}\right)-k_{w}w_{2}+k_{p}\nabla^{2}w_{2}\right)\delta w_{2}rdrd\theta$$

(46) $$\delta U=\underset{v}{∭}\sigma_{1}{}_{ij}^{NL}\delta \varepsilon_{1}{}_{ij}dv+\underset{v}{∭}\sigma_{2}{}_{ij}^{NL}\delta \varepsilon_{2}{}_{ij}dv\;\;\;\;\;i,j=r,\theta$$

(47) $$\delta \Pi =\delta U+\delta \Omega_{1}+\delta \Omega_{2}=0$$

where the upper layer and the lower layer are numbered 1 and 2, respectively. The equilibrium equations in terms of local stresses for the first layer (*i* = 1) and the second layer (*i* = 2) are described as follows.

(48) $$\delta u_{i}:Ni_{r}^{L}+r\frac{dNi_{r}^{L}}{dr}-Ni_{\theta }^{L}=0\;,\;i=1,2$$

(49) $$\begin{array}{l} \delta w_{1}:r\frac{d^{2}M_{1}{}_{r}^{L}}{dr^{2}}+2\frac{dM_{1}{}_{r}^{L}}{dr}-\frac{dM_{1}{}_{\theta }^{L}}{dr}+\left(1-\mu \nabla^{2}\right)\left(\left(q+k_{0}\left(w_{2}-w_{1}\right)\right)r\right.+\\ N_{1}{}_{\theta }^{L}\frac{dw_{1}}{dr}+\left.rN_{1}{}_{r}^{L}\frac{d^{2}w_{1}}{dr^{2}}\right)+\left.\mu r\left(\left(\nabla^{2}N_{1r}^{L}\right)\frac{d^{2}w_{1}}{dr^{2}}+\left(\nabla^{2}N_{1\theta }^{L}\right)\frac{1}{r}\frac{dw_{1}}{dr}\right.\right)=0 \end{array}$$

(50) $$\begin{array}{l} \delta w_{2}:r\frac{d^{2}M_{2}{}_{r}^{L}}{dr^{2}}+2\frac{dM_{2}{}_{r}^{L}}{dr}-\frac{dM_{2}{}_{\theta }^{L}}{dr}+\left(1-\mu \nabla^{2}\right)\left(\left(-k_{0}\left(w_{2}-w_{1}\right)-k_{w}w_{2}+k_{p}\nabla^{2}w_{2}\right)r\right.+\\ N_{2}{}_{\theta }^{L}\frac{dw_{2}}{dr}+\left.rN_{2}{}_{r}^{L}\frac{d^{2}w_{2}}{dr^{2}}\right)+\mu r\left(\left(\nabla^{2}N_{2r}^{L}\right)\frac{d^{2}w_{2}}{dr^{2}}\right.+\left.\left(\nabla^{2}N_{2\theta }^{L}\right)\left(\frac{1}{r}\frac{dw_{2}}{dr}\right)\right)=0 \end{array}$$

(51) $$\delta \phi_{i}:y^{*}\left(r\frac{dMi_{r}^{L}}{dr}+Mi_{r}^{L}-Mi_{\theta }^{L}-rQi_{r}^{L}\right)+Ri_{r}^{L}+r\frac{dRi_{r}^{L}}{dr}-Ri_{\theta }^{L}-rRi_{rz}^{L}=0,\;i=1,2$$

### 2.4. Boundary Conditions

The boundary conditions of the circular/annular plate are as follows:

Simply supported (S):

(52) $$u=w=M_{r}=R_{r}=0$$

Clamped (C):

(53) $$u=w=\phi =\frac{dw}{dr}=0$$

Free (F):

(54) $$N_{r}=M_{r}=R_{r}=Q_{r}=0\;$$

### 2.5. Dimensionless Assumptions

Due to the small values in the nanoscale and for the simplicity of calculations, the following relations are used to make the governing equations of the nanoplate dimensionless:

(55) $$\begin{array}{l} u^{*}=\frac{u_{0}}{h};w^{*}=\frac{w_{0}}{r_{o}};\phi^{*}=\phi \;;\;\psi^{*}=\psi ;N_{r}^{*}=\frac{N_{r}}{E_{1}h};\;N_{\theta }^{*}=\frac{N_{\theta }}{E_{1}h};\\ \;Q_{r}^{*}=\frac{Q_{r}}{E_{1}h}\;;Q_{\theta }^{*}=\frac{Q_{\theta }}{E_{1}h};M_{r}^{*}=\frac{M_{r}}{E_{1}h^{2}}\;;\;M_{\theta }^{*}=\frac{M_{\theta }}{E_{1}h^{2}}\;;\;\nabla^{*}{}^{2}=\frac{d^{2}}{dr^{*}{}^{2}}+\frac{1}{r^{*}}\frac{d}{dr^{*}};\\ R_{r}^{*}=\frac{R_{r}}{E_{1}h^{2}};R_{\theta }^{*}=\frac{R_{\theta }}{E_{1}h^{2}};R_{rz}^{*}=\frac{R_{rz}}{E_{1}h};\;r^{*}=\frac{r}{r_{o}}\;;\;z^{*}=\frac{z}{h}\;;\;\\ \delta =\frac{h}{r_{o}};\;\mu^{*}=\frac{\mu }{r_{o}^{2}}\;;l^{*}=\frac{l}{r_{o}^{}};q^{*}=\frac{q}{E_{1}}\;;\;k_{w}^{*}=\frac{k_{w}r_{o}}{E_{1}}\;;k_{p}^{*}=\frac{k_{p}}{E_{1}r_{o}} \end{array}$$

## 3. Numerical Solution Method

The differential quadrature technique is one of the high-accuracy numerical methods, and it is derived from the quadratic integration method. In the quadratic integration method, the integral at one point along the domain depends on all points along that direction. The value of dependency is determined by weight coefficients.

(56) $$\int_{a}^{b}f(r)dr=\sum_{k=1}^{n}w_{k}f_{k}$$

In the above equation, $w_{1},w_{2},\ldots ,w_{n}$ are weight coefficients and $f_{1},f_{2},\ldots ,f_{n}$ are function values at discrete points. Regarding quadratic integration, Belman et al. [56] suggested that the derivative at one point of the function domain depends on the function values at all points of the domain by weight coefficients:

(57) $${\left.\frac{df}{dr}\right|}_{r_{i}}=\sum_{j=1}^{N}A_{ij}f\left(r_{j}\right)\;,\;i=1,2,\ldots ,N$$

In the above equation, $A_{ij}$ is the weight coefficient, and *N* is the total number of nodes in the direction of *r*. The weighting coefficients for the first-order derivative are obtained as follows:

(58) $$A_{ij}^{\left(1\right)}=\frac{P\left(r_{i}\right)}{\left(r_{i}-r_{j}\right)P\left(r_{j}\right)}$$

(59) $$P\left(r_{i}\right)=\overset{N}{\underset{j=1}{\prod }}\left(r_{i}-r_{j}\right)\;,\;i\neq j$$

(60) $$A_{ii}^{\left(1\right)}=-\sum_{k=1}^{N}A_{ik}^{\left(1\right)}\;,\;i\neq k$$

Additionally, higher-order derivatives are obtained as follows:

(61) $${\left.\frac{d^{\left(n\right)}f}{dr^{\left(n\right)}}\right|}_{r_{i}}=\sum_{j=1}^{N}A_{ij}^{\left(n\right)}f(r_{j})\;,\;i=1,\ldots ,N$$

The weighting coefficients for derivatives of the second and higher orders are introduced in the form of the following equations:

(62) $$A_{ij}^{\left(n\right)}=n\left[A_{ij}^{\left(1\right)}A_{ii}^{\left(n-1\right)}-\frac{A_{ij}^{\left(n-1\right)}}{\left(r_{i}-r_{j}\right)}\right]\;,\;i\neq j$$

(63) $$A_{ii}^{\left(n\right)}=-\sum_{j=1,\neq i}^{N}A_{ij}^{\left(n\right)}\;,\;i,j=1\ldots N$$

In this paper, the distribution of grid points based on Chebyshev–Gauss–Lubato points is used, which enhances the convergence speed of the solution and is described in the following form:

(64) $$r_{i}=\frac{r_{i}+r_{o}}{2}-\cos\left(\left(\frac{i-1}{N-1}\right)\pi \right)\left(\frac{r_{o}-r_{i}}{2}\right),\;i=1\ldots N$$

In the above equation, $r_{i}$ and $r_{o}$ are the points at the beginning and end of the function, respectively.

## 4. Results and Discussion

In this section, different factors are investigated to observe how they influence the deflections of the circular/annular nanoplate based on FSDT and HSDT, and the nonlocal strain gradient model is considered via DQM. In addition, to validate the solution method, the present results (in the case of the circular/annular nanoplate subjected to bending loading loads) are compared with the results of references.

**Figure 4:**
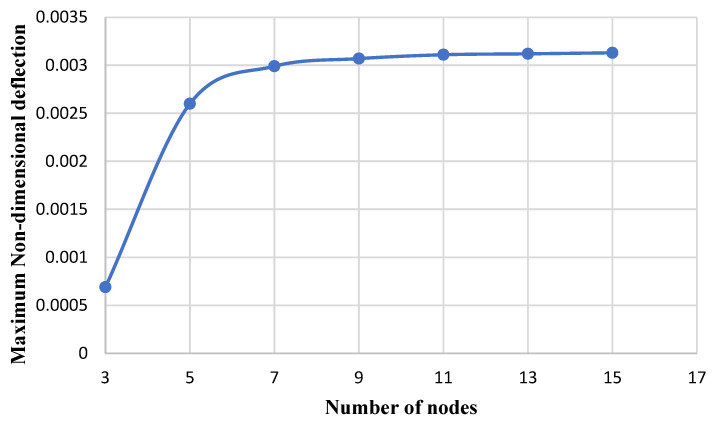
The variation of dimensionless maximum deflection versus the number of nodes.

Figure 4 shows the effect of the number of nodes used in the differential quadratic method on the results of the present study (the maximum dimensionless deflection of the circular/annular nano plate). As observed, after nine nodes, proper convergence is achieved. In this research study, the number of 11 nodes is used to calculate the results.

**Figure 5:**
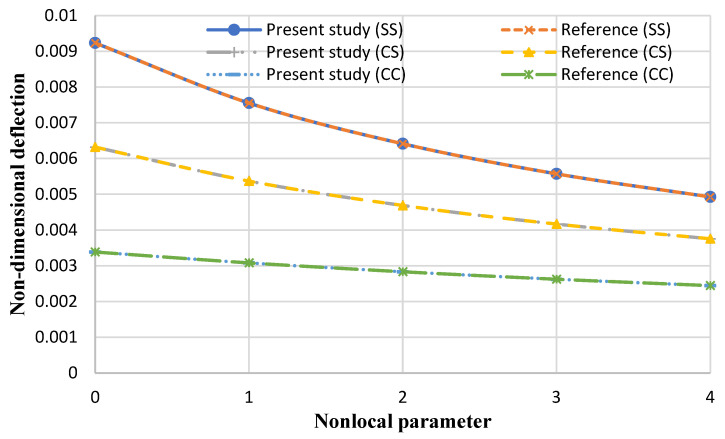
Comparison of the present numerical results with a reference for the maximum dimensionless deflection of the annular nanoplate versus the nonlocal coefficient.

To validate the current research and solution method, the results obtained have been compared with reference [57], and the results can be observed in Figure 5 for clamped (C) and simply supported (S) boundary conditions. In this reference, the FSDT is used, and the following values are considered:

(65) $$\begin{array}{l} E_{1}=1060\left(\mathrm{GPa}\right)\;,\;E_{2}=1060\left(\mathrm{GPa}\right)\;,\;\nu_{12}=0.3\;,\;\nu_{21}=0.3,\;q=0.1\;\left(\mathrm{GPa}\right)\;,\;k_{w}^{*}=0.004717\;,\\ \;k_{p}^{*}=0\;,\;h=0.34\;\left(\mathrm{nm}\right)\;,\;r_{o}=5\;\left(\mathrm{nm}\right)\;,\;r_{i}=1\;\left(\mathrm{nm}\right). \end{array}$$

Based on Figure 5, it can be observed that the results of the present paper are in good agreement with the reference. Furthermore, this figure illustrates that the flexibility of the nanoplate decreases with an increasing non-local coefficient [57].

Table 2 compares the deflection of the circular nanoplate with refs. [57,58,59,60] by considering the following assumptions:

(66) $$E_{1}=E_{2}=2\times 10^{6}\mathrm{Pa},\mu =0,\nu_{12}=\nu_{21}=0.3,R^{*}=\frac{r_{0}}{h}=10$$

As can be compared, the results of the present study are in good harmony with the results of the references.

Table 3 and Table 4 compare the dimensionless maximum deflection obtained from FSDT and HSDT by assuming different functions for a circular nanoplate in clamped and simply supported boundary conditions using different thickness-to-radius ratios (*α* = *h*/*r_o_*) and non-local coefficients *μ* and considering the following:

(67) $$\begin{array}{l} \;k_{w}^{*}=1.13\times 10^{9};k_{p}^{*}=1.13\times 10^{9},\;E_{1}=1765\;\mathrm{GPa},\;E_{2}=1588\;\mathrm{GPa},\\ \nu_{12}=0.3,\;\nu_{21}=0.27,\;h=0.34\;\mathrm{nm},q^{*}=1\times 10^{9} \end{array}$$

It can be observed in Table 3 and Table 4 that by increasing the nonlocal elasticity parameter, the nondimensional deflection is reduced. Moreover, it can be discerned that, by increasing the thickness-to-radius ratio, deflection decreases. It can be observed that for different nonlocal coefficients and thickness-to-radius ratios, the FSDT overestimates the results in comparison with the HSDT.

In this comparison, several different functions have been used as a function to distribute the shear stress along the thickness. These functions are as follows:

(68) $$g_{1}\left(z\right)=\frac{h}{\pi }\sin\left(\frac{\pi z}{h}\right)$$

(69) $$g_{2}\left(z\right)=-\frac{4}{3h^{2}}z^{3}+z$$

(70) $$g_{3}\left(z\right)=h\sinh\left(\frac{z}{h}\right)-z\cosh\left(\frac{1}{2}\right)$$

(71) $$g_{4}\left(z\right)=ze^{-2{\left(\frac{z}{h}\right)}^{2}}$$

(72) $$g_{5}\left(z\right)=-\frac{5}{3h^{2}}z^{3}+\frac{5}{4}z$$

As observed in Table 3 and Table 4, the results of using these functions in the HSDT have very little difference when compared to each other, and it can be concluded that using these various functions interchangeably does not result in a significant difference when calculating nanoscale bending. In fact, compared to other parameters (such as the effect of the nonlocal parameter or strain gradient coefficient), using various *g*(*z*) interchangeably has insignificant effects on the results. These functions assume the distribution of the shear stress along the thickness of the plate and satisfy the boundary condition of the shear stress at the top and bottom of the plate. Additionally, unlike the FSDT, there is no need to use a shear correction factor. From the tables, it is observed that deflection decreases with an increase in the order of the theory. At small thicknesses, the difference between the maximum dimensionless deflection of the FSDT and HSDT is almost negligible. As the ratio of the thickness to radius (*h*/*r*) increases, the difference between the results of the two theories increases. This significant difference indicates that, on thick plates, more accurate results are obtained by using HSDT. On the other hand, as the nonlocal coefficient is enhanced, the difference between the results of the two theories increases (but this factor is not more significant than the effect of *h*/*r*).

**Figure 6:**
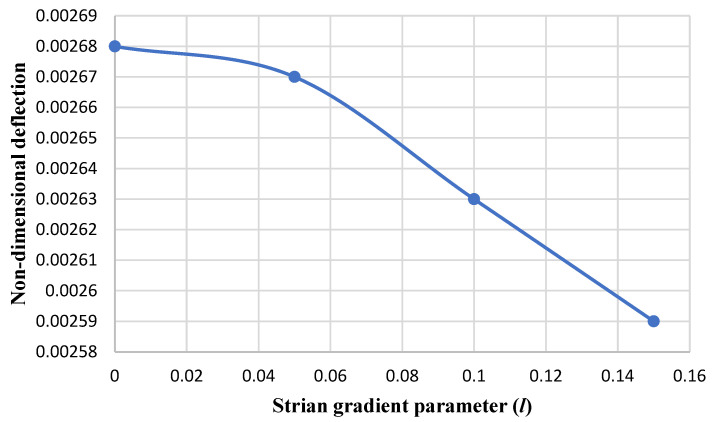
The maximum nondimensional deflection of the circular nanoplate versus the strain gradient parameter (*l*) in the clamped boundary condition.

**Figure 7:**
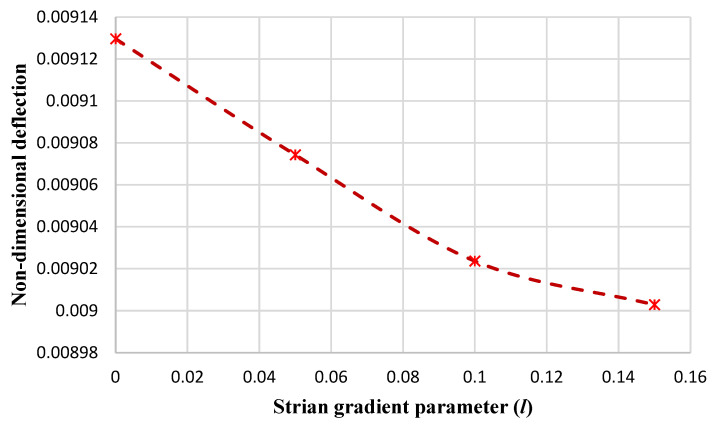
The maximum nondimensional deflection of the circular nanoplate versus the strain parameter (*l*) in the simply supported boundary condition.

Figure 6 and Figure 7 illustrate the results of the nondimensional maximum deflection (obtained from HSDT) versus the strain gradient parameter for the circular nanoplate at the clamped and simply supported boundary conditions, respectively. It can be observed that with the enhancement of the strain coefficient, the maximum deflection of the nanoplate decreases. This can be persuaded: by increasing the strain coefficient, the stiffness of the nanoplate is enhanced, so as a result, the deflection of the plate is reduced, which is in agreement with the results in paper [14].

Table 5 compares the results of FSDT with the average results obtained from HSDT in the clamped boundary condition. It can be seen that by increasing the nonlocal elasticity, the maximum deflection of the nanoplate decreases [57]. Additionally, the results considering different shape functions are almost the same.

**Figure 8:**
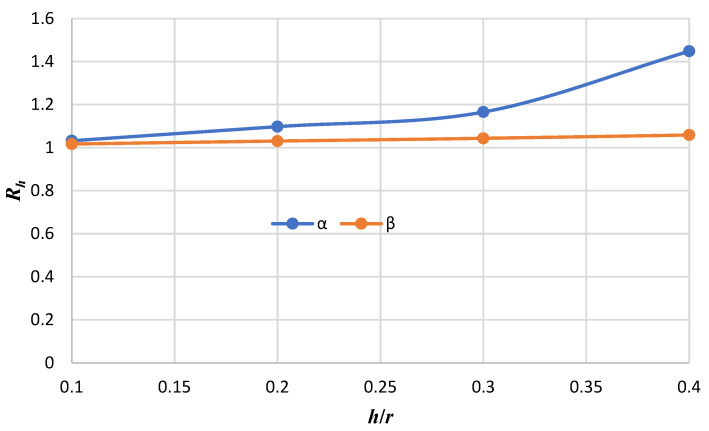
*R_h_* versus the thickness-to-radius ratio for *α* and *β* ratios in the clamped boundary condition.

Figure 8 shows the variation of $R_{h}$ (the ratio of the maximum dimensionless deflection obtained from FSDT to HSDT) in terms of *α* and *β* ratios for a circular nanoplate with the clamped boundary condition. $R_{h}$ can be defined as follows:

(73) $$R_{h}=\frac{w_{FSDT}^{*}}{w_{HSDT}^{*}}$$

where $w_{FSDT}^{*}$ and $w_{HSDT}^{*}$ are the ratios of the maximum dimensionless deflection obtained from FSDT and HSDT, respectively.

In the diagram, α is defined when thickness *h* is considered constant (*h* = 0.34 nm) and the radius changes; moreover, *β* is assumed when the radius is considered constant (*r* = 7 nm) and the thickness changes as follows:

(74) $$\alpha =\frac{h}{r},h\;\mathrm{constant}$$

(75) $$\beta =\frac{h}{r},r\;\mathrm{constant}$$

From the graph, it can be discerned that $R_{h}$ increases as the ratio of the thickness-to-radius increases; that is, the difference between the results of the two theories (FSDT and HSDT) is enhanced with an increasing thickness-to-radius ratio. It can be observed that at the same values of *h*/*r*, the differences between these theories are obtained with various *α* and *β* ratios. In other words, with the same *h*/*r*, the effect of changing the radius on the difference between the results of the two theories is greater than the effect of changing the thickness.

**Figure 9:**
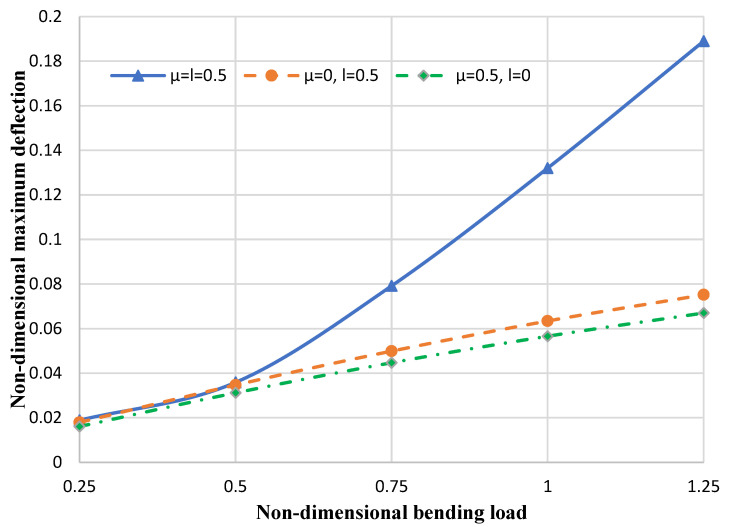
Nondimensional deflection of the circular nanoplate versus different load values.

Figure 9 reveals the nondimensional maximum deflection versus dimensionless bending loads for different nonlocal and strain gradient parameters in the clamped boundary condition. It can be concluded that both the strain gradient and nonlocal parameters have notable effects in reducing or increasing the dimensionless maximum deflection of the nanoplate. In addition, it is observed that with increasing load values, the importance of considering both strain gradient and nonlocal coefficients in the bending analyses of nanoplates is highlighted.

**Figure 10:**
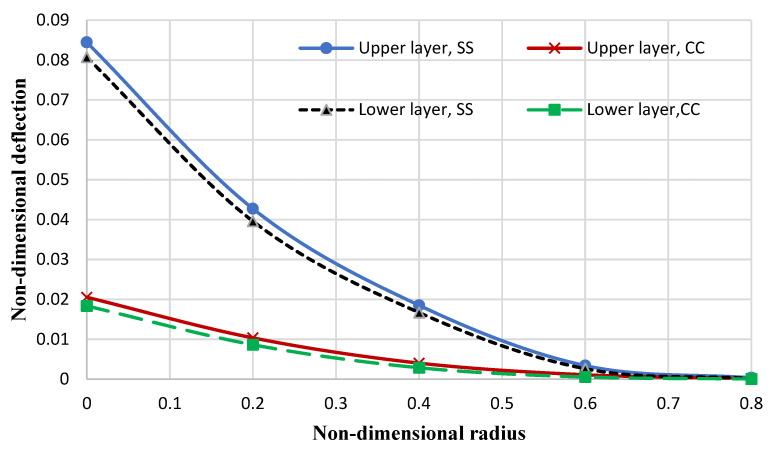
Nondimensional deflection of the annular bilayer nanoplate.

**Figure 11:**
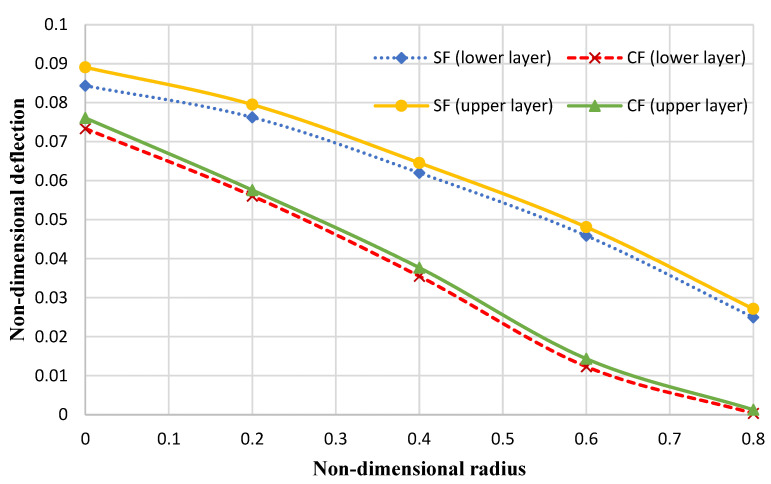
The nondimensional maximum deflection of the annular bilayer nanoplate.

Additionally, Figure 10 and Figure 11 depict the nondimensional deflection of the annular nanoplate by considering certain nonlocal elasticity and strain gradient coefficients at simply supported (S), clamped (C), and free (F) boundary conditions and by considering the following assumptions:

(76) $$\begin{array}{l} E_{1}=1765\;\left(\mathrm{GPa}\right),\;E_{2}=1588\;\left(\mathrm{GPa}\right),\;\nu_{12}=0.3,\;\nu_{21}=0.27,\;r_{o}=5\;\left(\mathrm{nm}\right),\;r_{i}=0.2r_{o},\;k_{p}=1.13\;\left(\mathrm{Pa}\cdot m\right),\\ \;k_{w}=1.13\;\left(\mathrm{GPa}/\mathrm{nm}\right),\;h=0.34\;\left(\mathrm{nm}\right),\;K_{o}=45\;\left(\mathrm{GPa}/\mathrm{nm}\right) \end{array}$$

It can be observed that an increase in the nondimensional radius results in a reduction in the nondimensional deflection at all boundary conditions for both layers. Additionally, it can be observed that in the simply supported condition, the deflection is more significantly reduced than in the clamped condition. In other words, the slope of the curve (of the deflection) decreases with decreasing plate flexibility.

**Figure 12:**
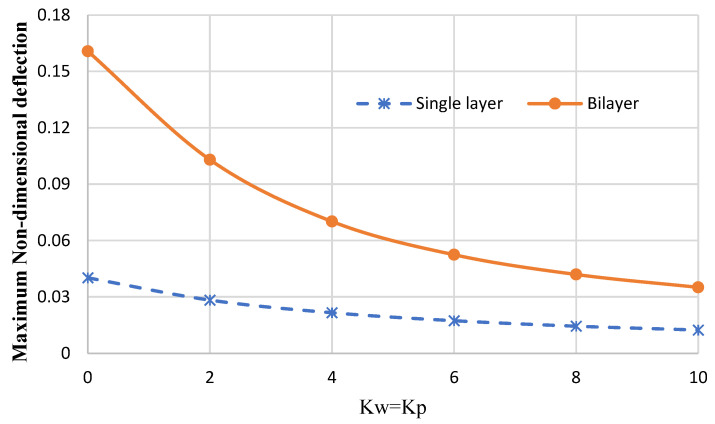
The variation of the nondimensional maximum deflection for the circular nanoplate for single-layer and bilayer nanoplates.

Figure 12 shows the variation of the maximum dimensionless deflection in terms of the Winkler and Pasternak elastic coefficients for the circular bilayer nanoplate (the thickness of each layer is 0.34 nm) and the single-layer nanoplate (with a thickness of 0.68 nm) for the clamped boundary condition by considering *r* = 5 nm, and *q* = 1 GPa.

In fact, this figure examines the possibility of replacing a bilayer nanoplate with a single-layer nanoplate. It is clear from the diagram that it is not possible to replace a bilayer nanoplate with a single-layer nanoplate (with the same thickness as the bilayer). In addition, the deflection of the single-layer nanoplate is lower than the deflection of a bilayer nanoplate. In this paper, the interaction between two monolayers of graphene plates is considered a result of the van der Waals interaction. The interpretation (of Figure 12) can be justified as the van der Waals interaction is a relatively weak force. If the bending loads (applied to the plate) are high enough, the molecules break free of the van der Waals forces that hold them together, so the results of a monolayer plate are not the same as the bilayer plate, especially under higher bending loads.

Additionally, it can be observed that, by increasing the stiffness of the elastic foundations, the results converge. In other words, increasing the stiffness of the elastic foundation (or reducing the bending loads) can lead to more similarities between the results obtained from the single-layer plate and the bilayer plate.

## 5. Conclusions

The nonlinear bending of the axisymmetric circular/annular nanoplate using the nonlocal strain gradient model with FSDT and HSDT is studied. Additionally, a bilayer circular/annular nanoplate is analyzed. To solve governing equations, DQM was used for the circular/annular nanoplate. For validation, the results were compared with other references. Some of the important results of this paper are as follows:

∗ Deflection decreases with the increasing order of the theory. In other words, FSDT overestimates the deflection results, especially at higher thickness-to-radius ratios, so it is better to use HSDT models in those cases.
∗ The ratio of the maximum dimensionless deflection obtained from FSDT to HSDT is enhanced as the ratio of the thickness-to-radius increases.
∗ With the increase in the strain gradient parameter, the nondimensional maximum deflection of the nanoplate decreases.
∗ Replacing a bilayer nanoplate (considering van der Waals forces between layers) with a single-layer nanoplate (which has the same equivalent thickness as the bilayer plate) is not possible when obtaining exact deflection results, especially when reducing the stiffness of the elastic foundations (or in higher bending loads). In addition, the single-layer nanoplate underestimates the deflection results of the bilayer nanoplate (with the same equivalent thickness as the single-layer nanoplate).

## Figures and Tables

**Table 1:** Some of the suggested functions for HSDT in different references.

Model	g(z) Function
Ambartsumian [47]	$-\frac{1}{6}z^{3}+\frac{h^{2}}{8}z$
Reddy [48]	$-\frac{4}{3h^{2}}z^{3}+z$
Reissner [49]	$-\frac{5}{3h^{2}}z^{3}+\frac{5}{4}z$
Touratier [50]	$\frac{h}{\pi }\sin\left(\frac{\pi z}{h}\right)$
Soldatos [51]	$h\sinh\left(\frac{z}{h}\right)-z\cosh\left(\frac{1}{2}\right)$
Aydogdu [52]	$ze^{-2{\left(\frac{z}{h}\right)}^{2}}$
Mantari [53]	$\frac{h}{\pi }\left(\sin\left(\frac{\pi z}{h}\right)e^{m\cos\left(\frac{\pi z}{h}\right)}+m\frac{\pi }{h}z\;\right)\;,\;m\geq 0$

**Table 2:** Comparison of the non-dimensional deflection obtained from the present study with the other references for the circular plate.

q*	Ref. [58]	Ref. [60]	Ref. [59]	DQM Ref. [57]	SAPM Ref. [57]	Present (FSDT)	Present (g_1_)	Present (g_2_)	Present (g_3_)	Present (g_4_)	Present (g_5_)
0.0001	0.1678	0.1687	0.1706	0.168	0.1685	0.1685	0.1732	0.1801	0.1793	0.1789	0.1801
0.0003	0.4583	0.4655	0.5119	0.4588	0.4642	0.4642	0.471	0.4863	0.4843	0.4835	0.4863
0.001	1.0509	1.0937	1.7069	1.0514	1.0557	1.0557	1.0708	1.0929	1.0899	1.0887	1.0929

**Table 3:** Comparison of the dimensionless maximum deflection obtained from FSDT with HSDT (considering different functions) for a circular nanoplate under the clamped boundary condition.

*α* = *h*/*r_o_*	Theory	*μ* = 0	*μ* = 1	*μ* = 2	*μ* = 3	*μ* = 4
0.05	FSDT	0.09482	0.08832	0.08238	0.07706	0.07231
	HSDT-*g*_1_(*z*)	0.09413	0.08754	0.08145	0.07603	0.07125
	HSDT-*g*_2_(*z*)	0.09413	0.08753	0.08145	0.07603	0.07126
	HSDT-*g*_3_(*z*)	0.09412	0.08752	0.08144	0.07604	0.0713
	HSDT-*g*_4_(*z*)	0.09413	0.08754	0.08145	0.07604	0.07128
	HSDT-*g*_5_(*z*)	0.09413	0.08754	0.08145	0.076	0.07126
0.1	FSDT	0.06384	0.05277	0.04528	0.03973	0.03541
	HSDT-*g*_1_(*z*)	0.06310	0.05157	0.04403	0.03855	0.03433
	HSDT-g_2_(z)	0.06311	0.05156	0.04402	0.03855	0.03432
	HSDT-*g*_3_(*z*)	0.06312	0.05157	0.04403	0.03854	0.03433
	HSDT-*g*_4_(*z*)	0.06310	0.05156	0.04401	0.03855	0.03432
	HSDT-*g*_5_(*z*)	0.06311	0.05155	0.044	0.03854	0.03433
0.3	FSDT	0.00515	0.00429	0.00368	0.00323	0.00287
	HSDT-*g*_1_(*z*)	0.00494	0.00386	0.00316	0.00268	0.00232
	HSDT-*g*_2_(*z*)	0.00494	0.00385	0.00316	0.00268	0.00233
	HSDT-*g*_3_(*z*)	0.00495	0.00386	0.00317	0.00267	0.00231
	HSDT-*g*_4_(*z*)	0.00493	0.00385	0.00315	0.00266	0.00234
	HSDT-*g*_5_(*z*)	0.00495	0.00386	0.00317	0.00268	0.00233

**Table 4:** Comparison of the dimensionless maximum deflection obtained from FSDT with HSDT (considering different functions) for a circular nanoplate in the simply supported boundary condition.

*α* = *h*/*r_o_*	Theory	*μ* = 0	*μ* = 1	*μ* = 2	*μ* = 3	*μ* = 4
0.05	FSDT	0.09956	0.09710	0.09365	0.08989	0.08610
	HSDT-*g*_1_(*z*)	0.09960	0.09703	0.09338	0.08945	0.08557
	HSDT-*g*_2_(*z*)	0.09959	0.09703	0.09337	0.08941	0.08556
	HSDT-*g*_3_(*z*)	0.09961	0.09702	0.09338	0.08944	0.08565
	HSDT-*g*_4_(*z*)	0.09960	0.09703	0.09337	0.08945	0.08556
	HSDT-*g*_5_(*z*)	0.09961	0.097	0.09336	0.08556	0.08945
0.1	FSDT	0.09391	0.08401	0.07546	0.06814	0.06190
	HSDT-*g*_1_(*z*)	0.09389	0.08352	0.07493	0.0677	0.06176
	HSDT-*g*_2_(*z*)	0.0939	0.08353	0.07492	0.06778	0.06177
	HSDT-*g*_3_(*z*)	0.09389	0.0835	0.07493	0.06778	0.06177
	HSDT-*g*_4_(*z*)	0.0938	0.08352	0.07494	0.06824	0.06178
	HSDT-*g*_5_(*z*)	0.09389	0.08351	0.07493	0.06778	0.06177
0.3	FSDT	0.01623	0.01309	0.01079	0.00918	0.00806
	HSDT-*g*_1_(*z*)	0.01597	0.01274	0.0106	0.00912	0.00801
	HSDT-*g*_2_(*z*)	0.01598	0.01274	0.01063	0.0091	0.008
	HSDT-*g*_3_(*z*)	0.01599	0.01273	0.01064	0.00913	0.00801
	HSDT-*g*_4_(*z*)	0.01596	0.01272	0.01062	0.00912	0.00799
	HSDT-*g*_5_(*z*)	0.01598	0.01274	0.01063	0.00913	0.008

**Table 5:** Comparison of different shape functions at the maximum deflection of the circular nanoplate.

*μ*	*g*_1_ (HSDT)	*g*_2_ (HSDT)	*g*_3_ (HSDT)	*g*_4_ (HSDT)	*g*_5_ (HSDT)
0	0.00488	0.00488	0.00487	0.00487	0.00488
1	0.00379	0.0038	0.0038	0.00378	0.00379
2	0.00311	0.00311	0.0031	0.0031	0.00309
3	0.00263	0.00263	0.0026	0.00262	0.00263
4	0.00227	0.00228	0.00228	0.00229	0.00228

## Data Availability

Not applicable.

## References

[1] Roudbari M.A., Jorshari T.D., Lü C., Ansari R., Kouzani A.Z., Amabili M. (2022). A review of size-dependent continuum mechanics models for micro- and nano-structures. Thin-Walled Struct..

[2] Ghayesh M.H., Farajpour A. (2019). A review on the mechanics of functionally graded nanoscale and microscale structures. Int. J. Eng. Sci..

[3] Xiao Y., Luo F., Zhang Y., Hu F., Zhu M., Qin S. (2022). A Review on Graphene-Based Nano-Electromechanical Resonators: Fabrication, Performance, and Applications. Micromachines.

[4] Farajpour A., Ghayesh M.H., Farokhi H. (2018). A review on the mechanics of nanostructures. Int. J. Eng. Sci..

[5] Thai H.-T., Vo T.P., Nguyen T.-K., Kim S.-E. (2017). A review of continuum mechanics models for size-dependent analysis of beams and plates. Compos. Struct..

[6] Su Y., Zhou Z. (2020). Electromechanical Analysis of Flexoelectric Nanosensors Based on Nonlocal Elasticity Theory. Micromachines.

[7] Gui Y., Wu R. (2023). Buckling analysis of embedded thermo-magneto-electro-elastic nano cylindrical shell subjected to axial load with nonlocal strain gradient theory. Mech. Res. Commun..

[8] Li Q., Wu D., Gao W., Hui D. (2023). Nonlinear dynamic stability analysis of axial impact loaded structures via the nonlocal strain gradient theory. Appl. Math. Model..

[9] Mohammadian M., Hosseini S.M. (2022). A size-dependent differential quadrature element model for vibration analysis of FG CNT reinforced composite microrods based on the higher order Love-Bishop rod model and the nonlocal strain gradient theory. Eng. Anal. Bound. Elem..

[10] Penna R., Feo L., Lovisi G. (2021). Hygro-thermal bending behavior of porous FG nano-beams via local/nonlocal strain and stress gradient theories of elasticity. Compos. Struct..

[11] Cuong-Le T., Nguyen K.D., Hoang-Le M., Sang-To T., Phan-Vu P., Wahab M.A. (2022). Nonlocal strain gradient IGA numerical solution for static bending, free vibration and buckling of sigmoid FG sandwich nanoplate. Phys. B Condens. Matter.

[12] Karami B., Janghorban M., Rabczuk T. (2019). Static analysis of functionally graded anisotropic nanoplates using nonlocal strain gradient theory. Compos. Struct..

[13] Alghanmi R.A. (2022). Nonlocal Strain Gradient Theory for the Bending of Functionally Graded Porous Nanoplates. Materials.

[14] Arefi M., Kiani M., Rabczuk T. (2019). Application of nonlocal strain gradient theory to size dependent bending analysis of a sandwich porous nanoplate integrated with piezomagnetic face-sheets. Compos. Part B Eng..

[15] Bera P., Varun J.P., Mahato P.K. (2022). Buckling analysis of isotropic and orthotropic square/rectangular plate using CLPT and different HSDT models. Mater. Today Proc..

[16] Zhong R., Hu S., Wang Q., Qin B., Shuai C. (2023). Legendre-meshfree vibration analysis of cross-ply laminated elliptical shell of revolution considering the effect of drop-off ply. Thin-Walled Struct..

[17] Sobhani E., Koohestani M., Civalek Ö., Avcar M. (2023). Natural frequency investigation of graphene oxide powder nanocomposite cylindrical shells surrounded by Winkler/Pasternak/Kerr elastic foundations with a focus on various boundary conditions. Eng. Anal. Bound. Elem..

[18] Nguyen V.D., Phung V.B. (2023). Static bending, free vibration, and buckling analyses of two-layer FGM plates with shear connectors resting on elastic foundations. Alex. Eng. J..

[19] Padawale N., Patil S., Datar G. (2023). Nonlinear vibration of annular radially graded plate subjected to temperature at one edge. Mater. Today Proc..

[20] Qin X., Shen Y., Chen W., Yang J., Peng L.X. (2021). Bending and free vibration analyses of circular stiffened plates using the FSDT mesh-free method. Int. J. Mech. Sci..

[21] He X.C., Yang J.S., Mei G.X., Peng L.X. (2022). Bending and free vibration analyses of ribbed plates with a hole based on the FSDT meshless method. Eng. Struct..

[22] Xu L.-L., Chen C.-P., Zheng Y.-F. (2022). Two-degrees-of-freedom nonlinear free vibration analysis of magneto-electro-elastic plate based on high order shear deformation theory. Commun. Nonlinear Sci. Numer. Simul..

[23] Kharghani N., Guedes Soares C. (2022). Application of layerwise HSDT and fracture analysis in the ultimate strength of composite plates with delamination in bending. Int. J. Solids Struct..

[24] Pavan G.S., Muppidi H., Dixit J. (2022). Static, free vibrational and buckling analysis of laminated composite beams using isogeometric collocation method. Eur. J. Mech. A/Solids.

[25] Rodrigues D.E.S., Belinha J., Dinis L.M.J.S., Natal Jorge R.M. (2021). The bending behaviour of antisymmetric cross-ply laminates using high-order shear deformation theories and a Radial Point Interpolation Method. Structures.

[26] Zghal S., Dammak F. (2021). Vibration characteristics of plates and shells with functionally graded pores imperfections using an enhanced finite shell element. Comput. Math. Appl..

[27] Trabelsi S., Frikha A., Zghal S., Dammak F. (2018). Thermal post-buckling analysis of functionally graded material structures using a modified FSDT. Int. J. Mech. Sci..

[28] Zghal S., Trabelsi S., Frikha A., Dammak F. (2021). Thermal free vibration analysis of functionally graded plates and panels with an improved finite shell element. J. Therm. Stress..

[29] Zghal S., Frikha A., Dammak F. (2020). Large deflection response-based geometrical nonlinearity of nanocomposite structures reinforced with carbon nanotubes. Appl. Math. Mech..

[30] Zghal S., Frikha A., Dammak F. (2018). Non-linear bending analysis of nanocomposites reinforced by graphene-nanotubes with finite shell element and membrane enhancement. Eng. Struct..

[31] Aghababaei R., Reddy J.N. (2009). Nonlocal third-order shear deformation plate theory with application to bending and vibration of plates. J. Sound Vib..

[32] Zghal S., Joueid N., Tornabene F., Dimitri R., Chrigui M., Dammak F. (2023). Time-Dependent Deflection Responses of FG Porous Structures Subjected to Different External Pulse Loads. J. Vib. Eng. Technol..

[33] Frikha A., Zghal S., Dammak F. (2018). Dynamic analysis of functionally graded carbon nanotubes-reinforced plate and shell structures using a double directors finite shell element. Aerosp. Sci. Technol..

[34] Zghal S., Frikha A., Dammak F. (2018). Free vibration analysis of carbon nanotube-reinforced functionally graded composite shell structures. Appl. Math. Model..

[35] Zghal S., Frikha A., Dammak F. (2017). Static analysis of functionally graded carbon nanotube-reinforced plate and shell structures. Compos. Struct..

[36] Frikha A., Zghal S., Dammak F. (2018). Finite rotation three and four nodes shell elements for functionally graded carbon nanotubes-reinforced thin composite shells analysis. Comput. Methods Appl. Mech. Eng..

[37] Zghal S., Trabelsi S., Dammak F. (2022). Post-buckling behavior of functionally graded and carbon-nanotubes based structures with different mechanical loadings. Mech. Based Des. Struct. Mach..

[38] Zghal S., Frikha A., Dammak F. (2018). Mechanical buckling analysis of functionally graded power-based and carbon nanotubes-reinforced composite plates and curved panels. Compos. Part B Eng..

[39] Qaderi S., Ghadiri M., Najafi M., Imam A., Soleimanimehr H. (2023). Size-dependent nonlinear vibration analysis of cracked graphene-platelets-reinforced-composites (GPLRC) plate under parametric excitation. Commun. Nonlinear Sci. Numer. Simul..

[40] Mahinzare M., Akhavan H., Ghadiri M. (2020). A nonlocal strain gradient theory for rotating thermo-mechanical characteristics on magnetically actuated viscoelastic functionally graded nanoshell. J. Intell. Mater. Syst. Struct..

[41] Rashidpour P., Ghadiri M., Zajkani A. (2020). Low-velocity impact analysis of viscoelastic composite laminated nanoplate based on nonlocal strain gradient theory for different boundary conditions. J. Sandw. Struct. Mater..

[42] Ghorbani K., Rajabpour A., Ghadiri M., Keshtkar Z. (2020). Investigation of surface effects on the natural frequency of a functionally graded cylindrical nanoshell based on nonlocal strain gradient theory. Eur. Phys. J. Plus.

[43] Szekrényes A. (2021). Application of differential quadrature method to delaminated first-order shear deformable composite plates. Thin-Walled Struct..

[44] Duryodhana D., Waddar S., Bonthu D., Pitchaimani J., Powar S., Doddamani M. (2023). Buckling and free vibrations behaviour through differential quadrature method for foamed composites. Results Eng..

[45] Han J., Li L., Jin G., Ma W., Feng J., Jia H., Chang D. (2018). Qualitative Identification of the Static Pull-In and Fundamental Frequency of One-Electrode MEMS Resonators. Micromachines.

[46] Liu B.L., Li S., Li Y.S. (2023). Bending of FGM sandwich plates with tunable auxetic core using DQM. Eur. J. Mech. A/Solids.

[47] Ambartsumian S. (1958). On the theory of bending plates. Izv. Otd. Tech. Nauk. AN SSSR.

[48] Reddy J.N. (1984). A simple higher-order theory for laminated composite plates. J. Appl. Mech. Dec..

[49] Reissner E. (1974). On tranverse bending of plates, including the effect of transverse shear deformation. Int. J. Solids Struct..

[50] Touratier M. (1991). An efficient standard plate theory. Int. J. Eng. Sci..

[51] Soldatos K. (1992). A transverse shear deformation theory for homogeneous monoclinic plates. Acta Mech..

[52] Aydogdu M. (2009). A new shear deformation theory for laminated composite plates. Compos. Struct..

[53] Mantari J., Oktem A., Soares C.G. (2012). A new higher order shear deformation theory for sandwich and composite laminated plates. Compos. Part B Eng..

[54] Lim C.W., Zhang G., Reddy J.N. (2015). A higher-order nonlocal elasticity and strain gradient theory and its applications in wave propagation. J. Mech. Phys. Solids.

[55] Li Q., Wu D., Chen X., Liu L., Yu Y., Gao W. (2018). Nonlinear vibration and dynamic buckling analyses of sandwich functionally graded porous plate with graphene platelet reinforcement resting on Winkler–Pasternak elastic foundation. Int. J. Mech. Sci..

[56] Bellman R., Casti J. (1971). Differential quadrature and long-term integration. J. Math. Anal. Appl..

[57] Dastjerdi S., Jabbarzadeh M., Aliabadi S. (2016). Nonlinear static analysis of single layer annular/circular graphene sheets embedded in Winkler–Pasternak elastic matrix based on non-local theory of Eringen. Ain Shams Eng. J..

[58] Altekin M., Yükseler R.F. Large Deflection Analysis of Clamped Circular Plates. Proceedings of the World Congress on Engineering.

[59] Szilard R. (1974). Theory and Analysis of Plates: Classical and Numerical Methods (Book).

[60] Timoshenko S., Woinowsky-Krieger S. (1959). Theory of Plates and Shells.

